# Physiological, hematological and biochemical factors associated with high-altitude headache in young Chinese males following acute exposure at 3700 m

**DOI:** 10.1186/s10194-018-0878-7

**Published:** 2018-07-25

**Authors:** Kun Wang, Menghan Zhang, Yi Li, Weilin Pu, Yanyun Ma, Yi Wang, Xiaoyu Liu, Longli Kang, Xiaofeng Wang, Jiucun Wang, Bin Qiao, Li Jin

**Affiliations:** 1 State Key Laboratory of Genetic Engineering, Collaborative Innovation Center for Genetics and Development, School of Life Sciences, Fudan University, Shanghai, 200438 China; 2 Ministry of Education Key Laboratory of Contemporary Anthropology, Department of Anthropology and Human Genetics, School of Life Sciences, Fudan University, Shanghai, 200438 China; 3 Human Phenome Institute, Fudan University, Shanghai, 201203 China; 4 Institute of Cardiovascular Disease, General Hospital of Jinan Military Region, Jinan, 250022 Shandong China; 5 Key Laboratory of High Altitude Environment and Genes Related to Diseases of Tibet Autonomous Region, School of Medicine, Xizang Minzu University, Xianyang, 712082 China; 6 Six Industrial Research Institute, Fudan University, Shanghai, 200433 China

**Keywords:** High-altitude headache, Hypoxia, Blood urea nitrogen, Oxygen saturation, Heart rate

## Abstract

**Background:**

High-altitude headache (HAH) is the most common sickness occurred in healthy people after rapid ascending to high altitude, and its risk factors were still not well understood. To investigate physiological, hematological and biochemical risk factors associated with high-altitude headache (HAH) after acute exposure to 3700 m, we conducted a two-stage, perspective observational study. In 72 h, total 318 young Han Chinese males ascended from sea level (altitude of 50 m) to altitude of 3700 m by train. Demographic data, physiological, hematological and biochemical parameters of all participants were collected within one week prior to the departure, and within 24 h after arrival.

**Results:**

The incidence of HAH was 74.84%. For parameters measured at sea level, participants with HAH exhibited significantly higher age and lower BUN (*p* < 0.05). For parameters measured at 3700 m, participants with HAH exhibited significantly lower blood oxygen saturation (SpO_2_), higher resting heart rate (HR), higher systolic blood pressure at resting (SBP) and lower blood urea nitrogen (BUN) (all p < 0.05). At 3700 m, the severity of HAH associated with SpO_2_, HR and BUN significantly (all p < 0.05). Multivariate logistic regression revealed that for parameters at sea level, BUN was associated with HAH [BUN (OR:0.77, 95% CI:0.60–0.99)] and for parameters at 3700 m, SpO_2_, HR and BUN were associated with HAH independently [SpO_2_ (OR:0.84, 95% CI:0.76–0.93); HR (OR:1.03, 95% CI:1.00–1.07); BUN (OR:0.64, 95% CI:0.46–0.88)]. No association between hematological parameters and HAH was observed.

**Conclusion:**

We confirmed that higher HR, lower SpO_2_ are independent risk factors for HAH. Furthermore, we found that at both 50 m and 3700 m, lower BUN is a novel independent risk factor for HAH, providing new insights for understanding the pathological mechanisms.

**Electronic supplementary material:**

The online version of this article (10.1186/s10194-018-0878-7) contains supplementary material, which is available to authorized users.

## Background

For lowlanders who rapidly ascend to altitude above 2500 m, headache has been considered as the most frequent complaint [1, 2]. According to the most widely-accepted diagnose criteria, the Lake Louise Consensus scoring system identified headache as the cardinal symptom of acute mountain sickness (AMS) [3]. The International Headache Society defined high altitude headache (HAH) as a headache that develops within 24 h of ascent to high altitude and resolves within 8 h of descent [4, 5]. Previous studies have reported that the incidence of HAH is over 70% within 24 h after rapidly ascending above 2500 m [2].

Numerous studies have been emerged on epidemiology, clinical characteristics, pathophysiological mechanisms, treatment and risk factors of HAH [1, 6–8]. Recent studies suggested that the cause of HAH may be hypoxia-induced cerebral cytotoxic oedema, brain swelling and increased intracranial pressure [6]. In addition to oxygen inhalation, aspirin and acetaminophen were often used for HAH treatment, and the effect are contradictory [9, 10]. Several studies have demonstrated that young age, smoking history, higher body mass index history of migraine, high heart rate (HR) and low pulse oxygen saturation (SpO_2_) are independent risk factors for HAH [1, 11–13]. Previously, Huang et al. performed the first investigation on the relationship between hematological parameters and HAH before and after rapid ascending at 3700 m with 45 subjects, and found that HAH is associated with sea-level reticulocyte and neutrophil counts [14]. Because erythrocytes are the principal carrier of oxygen in the circulatory system, the hematological parameters may provide useful information regarding HAH. Moreover, some studies demonstrated that fluid retention is an important feature of AMS, but other studies have demonstrated that low fluid intake is an independent risk factor of HAH, and glomerular filtration rate estimates increases with AMS severities after rapid ascent to high altitude [15–18]. Over all, most of the studies had small sample sizes, the results were contradictory, and no definitive clear answer is available.

The present study was based on the hypothesis that some hematological and biochemical parameters would be related with HAH. We aimed to explore the association between physiological, hematological, biochemical parameters (including renal function parameters) and the risk of HAH after a 3-days ascending to the altitude of 3700 m. We carried out a repeated measurement design based on two phases (50 m and 3700 m) before and after ascending at high altitude for 318 healthy young Han Chinese males. Physical, hematological and biochemical parameters were collected at each phase, respectively.

## Methods

### Participants

In total, 318 young Chinese males who lived at 50 m and ascended to Tibet for physical training were recruited in this study. All participants reported their disease history, medication history, smoking and drinking history in structured case report forms (CRFs). The inclusion criteria were healthy 18–35 year old Han Chinese men whose primary residence was at an altitude of ≤1000 m and had no high-altitude exposure in recent 2 years. The exclusion criteria were cardio-cerebrovascular diseases, neurological and psychiatric diseases, episodic or chronic migraine diseases or chronic headache symptoms (any headache occurring on more than 2 weeks/mouth), autoimmune diseases, respiratory diseases, malignancy, liver and kidney dysfunction, active infection or a bad cold. Participants who took acetazolamide, diuretics, steroids or nonsteroidal anti-inflammatory drugs during the ascending were excluded. Subjects who agreed to participate underwent a short instruction and explanation of the purpose of this study, and all participants have signed the informed consent before their first examinations. The study was approved by the Human Ethics Committee of Fudan University.

### Study procedures and measurements

All participants ascended to altitude of 3700 m (Lhasa, Tibet) within 72 h by train from sea level (altitude of 50 m, Henan). The baseline physiological, hematological and biochemical measurements were performed in the morning, one week prior to the departure at 50 m. Within 24 h after their arrival at 3700 m, the participants underwent assessments of their physiological, hematological and biochemical parameters, as well as HAH. All participants were monitored by trained physicians for any signs of high-altitude cerebral or pulmonary edema, and immediate treatment will be addressed for emergent cases [19]. During the period of study, all participants maintained the same diet. Caffeine beverage consumption, alcohol consumption and medication use were prohibited, smoking and heavy exercises or physical labor were also avoided.

Structured case report forms (CRFs) were used to record the demographic data (age, body mass index (BMI), chest circumstance, smoking and drinking history) and all measurements at sea level and 3700 m for each participants. After the acute exposure at 3700 m, the physicians scored HAH based on self-description of patients (0 = no headache; 1 = mild headache; 2 = moderate headache; 3 = severe headache) was recorded, and the time and place of headache onset was recorded retrospectively. We used a two-repeated measurement method to collect the heart rate at resting (HR, beats/min), systolic blood pressures at resting (SBP, mmHg), diastolic blood pressure at resting (DBP, mmHg) and Oxygen saturation (SpO_2_, %), operated twice by two independent professional physicians and recorded the average value. SBP and DBP were measured using a standardized mercury sphygmomanometer, while SpO_2_ was measured using Nellcor NPB-40 (USA).

Morning fasting venous blood (4 ml) was collected with EDTA-K2 at both sea level and 3700 m, and 2 ml of the samples were used to assay blood cell parameters by a hematology analyzer (Sysmex pocH-100i, Japan) within 2 h. The hematological parameters included red blood cell count (RBC, × 10^12^/L), hemoglobin (Hgb, g/L), hematocrit (Hct, %), mean corpuscular volume (MCV, fL), mean corpuscular hemoglobin (MCH, pg), white blood cell count (WBC, × 10^9^/L), lymphocyte percentage (LYM%), absolute lymphocyte count (LYM#), platelet count (PLT, × 10^9^/L) and mean platelet volume (MPV, fL). The rest 2 ml blood specimens were centrifuged 3000 r/min for 10 minus to separate plasma. Biochemical parameters including alanine aminotransferase (ALT, U/L), aspartate aminotransferase (AST, U/L), blood urea nitrogen (BUN, mmol/L), serum creatinine (CREA, umol/L), total serum bilirubin (T-BIL, umol/L), direct serum bilirubin (D-BIL, umol/L) and indirect serum bilirubin (I-BIL, umol/L) were measured using automatic biochemistry analyzer (TOSHIBA TBA-120FR).

The diagnosis of HAH was based on the International Classification of Headache Disorders 3β criteria [5], but not strictly (no test for descending), restricted by the complex situations of field study.

### Statistical analyses

**Fig. 1:**
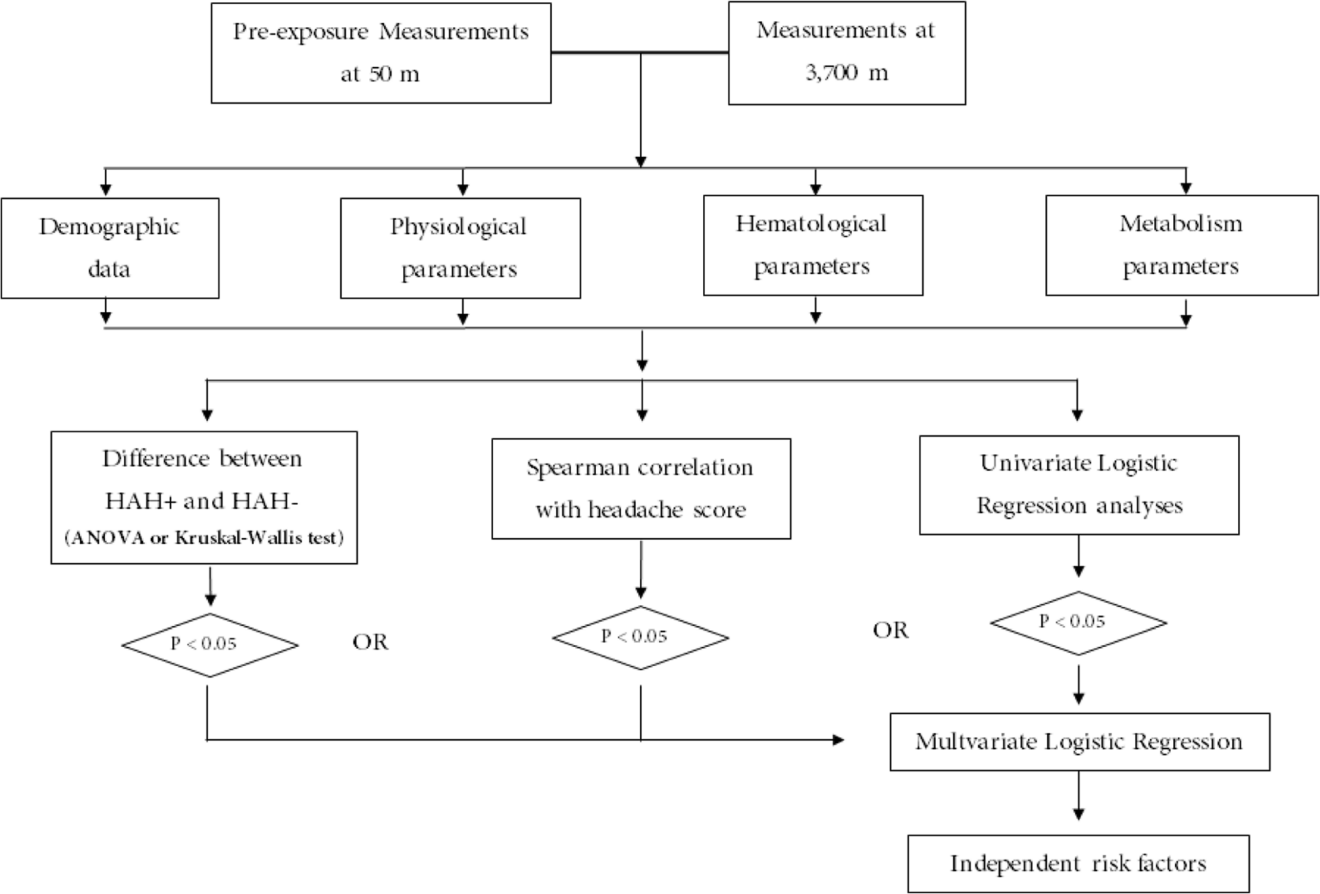
The flowchart of this study. HAH+, high-altitude headache (HAH); HAH-, none-HAH

The normality of continuous data was assessed by Shapiro-Wilk’s test. Normally distributed data were presented as the means±standard deviations (SD), non-normally distributed data were presented as median (interquartile range, IQR) and enumerated data were expressed as numbers (%). The differences of measurements between sea level and 3700 m were compared using paired-sample student’s t-test for normally distributed data, and the comparisons of differences between HAH positive (HAH+) and HAH negative (HAH-) groups were analyzed by independent student’s t-test. The non-normally data were compared using the Mann-Whitney U test. The spearman correlations between HAH score and the measurements at sea level and 3700 m were analyzed. Univariate logistic regression was performed to assess the relationships between each measurements and HAH at 3700 m. Significant variables in any of the above three analyses were included in forward stepwise multivariable logistic regression, and analyzed separately at sea level and 3700 m. The significant level of *p*-value is 0.05 (two-tailed). The analyses flowchart was shown in Fig. 1.

## Results

Total 318 participants had complete CRFs, physiological, hematological and biochemical measurements (Additional file 1). The mean age and BMI of the participants in this study were 21.87 ± 3.33 years and 22.06 ± 1.99 kg/m^2^. The incidence of HAH after acute exposure to 3700 m is 74.84% (Additional files 2 and 3).

### Alterations in physiological, hematological and biochemical measurements

Most of the physiological, hematological and biochemical parameters were dramatically altered after acute exposure to high altitude from sea level, beside of Hct, MCH and LYM%. For the physiological measurements, SpO_2_ decreased from 98.00 (IQR, 98.00–98.20) to 88.00 (IQR, 85.10–90.20) (*p* < 0.01), while HR, SBP and DBP significantly increased from sea level to 3700 m. For the hematological measurements, RBC, Hgb, Hct, PLT, MCH, MPV, WBC and LYM# all significantly increased, while MCV showed significant decrease (all *p* < 0.001). However, the alteration of LYM% between sea level and 3700 m was not significant. For the biochemical measurements, ALT, AST, CREA, TBIL, DBIL and IBIL exhibited a significant increase, while BUN had no significant difference between sea level and 3700 m. (Table 1).

**Table 1:** Comparison of physiological, hematological and biochemical parameters between sea level and 3700 m (*N* = 318)

	Measurements at sea-level	Measurements at 3700 m	*p*
Demographic data			
Age, y	21.87 ± 3.33	the same as sea-level	–
BMI, kg/m^2^	22.06 ± 1.99	the same as sea-level	–
chest circumstance, cm	86.11 ± 5.09	the same as sea-level	–
Smoking, yes(%)	102 (32.1)	the same as sea-level	–
Drinking, yes(%)	39 (12.3)	the same as sea-level	–
Physical parameters			
SpO_2_, %	98.00 (98.00–98.20)	88.00 (85.10–90.20)	< 0.001**
HR, beats/min	67.49 ± 9.18	84.17 ± 12.15	< 0.001**
SBP, mmHg	111.22 ± 9.81	120.52 ± 12.8	< 0.001**
DBP, mmHg	71.85 ± 8.14	81.77 ± 9.56	< 0.001**
Hematological parameters			
RBC, *10^12^	5.00 (4.80–5.20)	5.11 (4.82–5.49)	< 0.001**
Hgb, g/L	151.10 (144.00–157.00)	157.00 (147.00–169.30)	< 0.001**
Hct, %	44.49 ± 3.10	48.42 ± 6.08	< 0.001**
MCV, fL	92.20 (90.00–94.22)	86.50 (84.40–88.62)	< 0.001**
MCH, pg	30.59 (29.61–31.30)	30.90 (30.20–32.10)	< 0.001**
PLT, *10^9^	206.79 ± 44.77	244.97 ± 69.80	< 0.001**
MPV, fL	10.50 (9.80–11.00)	10.60 (10.20–11.00)	0.006**
WBC, *10^9^	6.10 (5.40–7.10)	7.90 (7.00–9.50)	< 0.001**
LYM%, %	36.23 ± 7.59	36.49 ± 9.06	0.833
LYM#, *10^9^	2.20 (1.90–2.60)	2.95 (2.50–3.40)	< 0.001**
Biochemical parameters			
ALT, U/L	18.00 (15.00–23.00)	39.20 (30.40–48.50)	< 0.001**
AST, U/L	15.10 (12.30–18.50)	31.00 (25.50–40.60)	< 0.001**
BUN, mmol/L	4.45 (3.89–5.28)	4.60 (4.10–5.25)	0.109
CREA, umol/L	58.00 (49.00–68.10)	105.40 (98.60–114.00)	< 0.001**
TBIL, umol/L	12.60 (10.80–14.00)	12.30 (10.20–15.65)	0.023*
DBIL, umol/L	3.10 (2.30–3.50)	4.70 (3.90–5.80)	< 0.001**
IBIL, umol/L	9.70 (8.50–10.50)	7.70 (5.80–10.65)	< 0.001**

*SpO*_*2*_ blood oxygen saturation, *HR* heart rate at resting, *SBP* systolic blood pressure, *DBP* diastolic blood pressure, *RBC* red blood cell count, *Hgb* hemoglobin, *Hct* hematocrit, *MCV* mean corpuscular volume, *MCH* mean corpuscular hemoglobin, *PLT* platelets count, *MPV* mean platelet volume, *WBC* white blood cell, *LYM%* lymphocyte rate, *LYM#* lymphocyte count, *ALT* alanine aminotransferase, *AST* aspartate aminotransferase, *BUN* blood urea nitrogen, *CREA* creatinine, *TBIL* total serum bilirubin, *DBIL* direct serum bilirubin, *IBIL* indirect serum bilirubin

Normally distributed variables were presented as mean ± SD, and compared using paired-sample T test; Non-normally distributed variables were presented as mean (interquartile range), and compared using Mann-Whitney U test

*p value indicates *p* < 0.05; **p value indicates *p* < 0.01

### Comparison of physiological, hematological and biochemical parameters between the HAH+ and HAH- groups

Regarding the measurements at sea level, compared to the HAH- group, the HAH+ group had significant higher age (HAH+: 22.05 ± 3.53 vs HAH-: 21.30 ± 2.69, *p* = 0.046) and significant lower BUN (HAH+: 4.38 (3.82–5.08) vs HAH-: 4.69 (3.99–5.53), *p* = 0.029). There were no significant differences in the physiological and hematological measurements at sea level (all *p* > 0.05, Table 2).

**Table 2:** Difference between HAH+ and HAH- subjects in demographics, physiological, hematological and biochemical parameters at sea level and 3700 m

	Measurements at sea level			Measurements at 3700 m		
	HAH+ (*n* = 238)	HAH- (*n* = 80)	*p*	HAH+ (*n* = 238)	HAH- (*n* = 80)	*p*
Demographic data						
Age (year)	22.05 ± 3.53	21.30 ± 2.69	0.046*	The same as sea-levels		
BMI (kg/m^2^)	22.08 ± 2.02	21.96 ± 1.86	0.662	The same as sea-levels		
Chest Circumstance (cm)	86.19 ± 5.30	86.04 ± 4.59	0.813	The same as sea-levels		
Smoking (yes, %)	73 (30.7)	29 (36.3)	0.417	The same as sea-levels		
Drinking (yes, %)	29 (12.2)	10 (12.5)	0.978	The same as sea-levels		
Physiological parameters						
SpO_2_ (%)	98.00 (97.90–98.00)	98.00 (98.00–98.00)	0.171	88.00 (84.90–90.40)	90.00 (87.30–92.10)	< 0.001**
HR (beats/min)	67.72 ± 8.99	66.91 ± 9.86	0.522	85.30 ± 12.09	80.77 ± 11.75	< 0.001**
SBP (mmHg)	110.97 ± 10.09	111.98 ± 8.88	0.405	119.74 ± 13.10	123.41 ± 11.10	0.046*
DBP (mmHg)	71.69 ± 8.23	72.30 ± 7.91	0.569	81.42 ± 9.86	82.99 ± 8.39	0.251
Hematological parameters						
RBC (*10^12^)	5.00 (4.80–5.20)	5.00 (4.70–5.20)	0.559	5.10 (4.83–5.48)	5.11 (4.75–5.55)	0.955
Hgb (g/L)	151.30 (144.00–157.00)	150.00 (143.80–158.10)	0.945	157.00 (149.60–168.30)	157.00 (144.50–170.40)	0.641
Hct (%)	45.69 ± 2.46	45.70 ± 2.49	0.981	48.61 ± 5.95	47.83 ± 6.47	0.364
MCV (fL)	92.30 (90.00–94.75)	92.00 (90.20–94.10)	0.598	86.50 (84.40–88.92)	86.40 (84.30–87.75)	0.336
MCH (pg)	30.60 (29.59–31.36)	30.59 (29.73–31.02)	0.459	30.90 (29.95–32.10)	31.00 (30.18–31.75)	0.939
PLT (*10^9^)	207.95 ± 44.97	203.33 ± 44.27	0.438	244.8 ± 68.6	245.3 ± 73.8	0.960
MPV (fL)	10.50 (9.80–11.02)	10.40 (9.70–10.91)	0.326	10.60 (10.20–11.03)	10.60 (10.30–11.00)	0.965
WBC (*10^9^)	6.00 (5.40–7.00)	6.50 (5.50–7.35)	0.090	7.90 (7.10–9.50)	7.95 (6.98–9.38)	0.791
LYM rate (%)	36.60 ± 7.58	35.12 ± 7.56	0.144	36.53 ± 9.68	36.36 ± 6.89	0.871
LYM count (*10^9^)	2.20 (1.90–2.60)	2.15 (1.90–2.60)	0.703	3.00 (2.51–3.40)	2.95 (2.58–3.50)	0.910
Biochemical parameters						
ALT (U/L)	18.10 (15.00–24.10)	18.00 (14.00–21.00)	0.187	39.20 (28.50–49.30)	39.00 (29.30–46.80)	0.563
AST (U/L)	15.20 (12.00–20.00)	14.00 (12.10–17.75)	0.237	31.00 (25.00–40.25)	32.00 (27.20–38.50)	0.581
BUN (mmol/L)	4.38 (3.82–5.08)	4.69 (3.99–5.53)	0.029*	4.50 (4.02–5.20)	4.80 (4.40–5.40)	0.003**
CREA (umol/L)	58.00 (49.00–65.00)	59.00 (50.10–68.20)	0.082	105.30 (98.50–115.00)	106.80 (99.70–113.30)	0.811
TBIL (umol/L)	12.50 (10.80–14.00)	12.70 (10.85–14.00)	0.876	12.75 (10.20–15.75)	12.90 (10.20–15.50)	0.709
DBIL (umol/L)	3.10 (2.30–3.50)	3.10 (2.15–3.50)	0.553	4.85 (3.90–5.90)	4.60 (3.80–5.80)	0.274
IBIL (umol/L)	9.60 (8.50–10.43)	9.90 (8.50–10.55)	0.933	7.60 (5.80–10.70)	7.80 (6.00–10.40)	0.877

*HAH* high altitude headache, *HAH+* with high-altitude headache, *HAH-* without high-altitude headache, *SpO*_*2*_ blood oxygen saturation, *HR* heart rate at resting, *SBP* systolic blood pressure, *DBP* diastolic blood pressure, *RBC* red blood cell count, *Hgb* hemoglobin, *Hct* hematocrit, *MCV* mean corpuscular volume, *MCH* mean corpuscular hemoglobin, *PLT* platelets count, *MPV* mean platelet volume, *WBC* white blood cell, *LYM%* lymphocyte rate, *LYM#* lymphocyte count, *ALT* alanine aminotransferase, *AST* aspartate aminotransferase, *BUN* blood urea nitrogen, *CREA* creatinine, *TBIL* total serum bilirubin, *DBIL* direct serum bilirubin, *IBIL* indirect serum bilirubin

Normally distributed variables were presented as mean ± SD, and compared using student’s t-test; Non-normally distributed variables were presented as mean (interquartile range), and compared using Mann-Whitney U test; Categorical variables were compared using fisher’s exact test;

*p value indicates *p* < 0.05; **p value indicates *p* < 0.01

Regarding the measurements at 3700 m, compared with the HAH- group, the HAH+ group had significant lower SpO_2_ (HAH+ 88.00 (84.90–90.40) vs HAH- 90.00 (87.30–92.10), *p* < 0.001), lower SBP (HAH+ 119.74 ± 13.10 vs HAH- 123.41 ± 11.10, p = 0.046) and lower BUN (HAH+ 4.50 (4.02–5.20) vs HAH- 4.80 (4.40–5.40), *p* = 0.003). The HAH+ group also had significant higher HR than HAH- group (HAH+ 85.30 ± 12.09 vs HAH- 80.77 ± 11.75, p < 0.001). The hematological measurements showed no significant differences between HAH+ and HAH- groups at 3700 m (all *p* > 0.05, Table 2).

### Relationship between physiological, hematological and biochemical parameters and HAH

We further used Spearman’s correlation analyses to explore the relationship between measurements and the HAH severity. For the measurements at sea level, no significant association was observed between demographic data or all measurements and HAH severity. For the measurements at 3700 m, HAH severity was significantly associated with SpO_2_ (*r* = − 0.365, *p* < 0.001), HR (*r* = 0.249, *p* = 0.002) and BUN (*r* = − 0.176, *p* = 0.006). The hematological measurements showed no significant correlation with HAH severity (all p > 0.05, Table 3).

**Table 3:** Relationships between HAH score and all the parameters at both sea level and 3700 m (*N* = 318)

	Measurements at sea-level		Measurements at 3700 m	
	With HAH score r	*p*	With HAH score r	*p*
Demographic data				
Age (y)	0.113	0.053	The same as sea-level	
BMI (kg/m^2^)	0.087	0.129	The same as sea-level	
Chest Circumstance (cm)	0.079	0.187	The same as sea-level	
Physical parameters				
SpO_2_ (%)	−0.085	0.168	−0.365	< 0.001**
HR (beats/min)	0.003	0.859	0.249	0.002**
SBP (mmHg)	−0.049	0.471	−0.050	0.518
DBP (mmHg)	−0.073	0.246	−0.008	0.865
Metabolic parameters				
RBC (*10^12^)	0.013	0.984	0.041	0.468
HGB (g/L)	0.008	0.910	0.046	0.358
HCT (%)	0.015	0.976	0.056	0.333
MCV (fL)	−0.012	0.927	−0.008	0.620
MCH (pg)	0.020	0.640	−0.042	0.941
PLT (*10^9^)	0.057	0.336	−0.021	0.420
MPV (fL)	0.077	0.428	−0.023	0.871
WBC (*10^9^)	−0.102	0.131	0.081	0.900
LYM% (%)	0.024	0.688	−0.061	0.341
LYM# (*10^9^)	0.035	0.687	0.017	0.278
Biochemical parameters				
ALT (U/L)	0.072	0.388	0.056	0.987
AST (U/L)	0.065	0.446	−0.051	0.100
BUN (mmol/L)	−0.071	0.211	−0.176	0.006**
CREA (umol/L)	−0.045	0.425	0.009	0.759
TBIL (umol/L)	−0.004	0.845	0.105	0.657
DBIL (umol/L)	0.037	0.581	0.034	0.672
IBIL (umol/L)	−0.027	0.539	0.106	0.583

*HAH* high altitude headache, *SpO*_*2*_ blood oxygen saturation, *HR* heart rate at resting, *SBP* systolic blood pressure, *DBP* diastolic blood pressure, *RBC* red blood cell count, *Hgb* hemoglobin, *Hct* hematocrit, *MCV* mean corpuscular volume, *MCH* mean corpuscular hemoglobin, *PLT* platelets count, *MPV* mean platelet volume, *WBC* white blood cell, *LYM%* lymphocyte rate, *LYM#* lymphocyte count, *ALT* alanine aminotransferase, *AST* aspartate aminotransferase, *BUN* blood urea nitrogen, *CREA* creatinine, *TBIL* total serum bilirubin, *DBIL* direct serum bilirubin, *IBIL* indirect serum bilirubin

*p value indicates *p* < 0.05; **p value indicates *p* < 0.01

### Risk factors for HAH at sea level and 3700 m

To discover risk factors for HAH, we performed univariate logistic regression for all parameters at sea level and at 3700 m. Among the sea level parameters, the univariate logistic regression revealed that only BUN associated with HAH significantly (OR:0.78, 95% CI:0.61–0.99, *p* = 0.044). Meanwhile, among the parameters at 3700 m, SpO_2_ (OR:0.84, 95% CI:0.77–0.90, *p* < 0.001), HR (OR:1.03, 95% CI:1.01–1.06, *p* = 0.004) and BUN (OR:0.72, 95% CI:0.55–0.93, *p* = 0.012) exhibited significant association with HAH (Table 4).

**Table 4:** Univariate logistic regression for each measurements at sea level and 3700 m (*N* = 318)

	Measurements at sea level				Measurements at 3700 m			
	β-coefficient	OR	95% CI	*p*	β-coefficient	OR	95% CI	*p*
Demographic Data								
age (y)	0.076	1.08	0.99–1.19	0.090	The same as sea level			
BMI (kg/m2)	0.028	1.03	0.90–1.18	0.674	The same as sea level			
Chest (cm)	0.006	1.01	0.96–1.06	0.825	The same as sea level			
smoking	−0.249	0.76	0.42–1.33	0.453	The same as sea level			
drinking	−0.055	0.97	0.45–2.16	0.917	The same as sea level			
Physical parameters								
SpO_2_ (%)	−0.058	0.94	0.75–1.12	0.555	− 0.176	0.84	0.77–0.90	< 0.001**
HR (beats/min)	0.010	1.01	0.98–1.04	0.501	0.032	1.03	1.01–1.06	0.004**
SBP (mmHg)	−0.010	0.99	0.96–1.02	0.433	−0.022	0.98	0.95–1.00	0.068
DBP (mmHg)	−0.009	0.99	0.96–1.02	0.576	−0.017	0.98	0.95–1.01	0.291
Hematological parameters								
RBC (*10^12^)	−0.197	0.82	0.39–1.76	0.605	0.072	1.07	0.75–1.55	0.696
HGB (g/L)	−0.002	1.00	0.97–1.03	0.906	0.004	1.00	0.99–1.02	0.460
HCT (%)	−0.001	1.00	0.90–1.11	0.981	0.022	1.02	0.98–1.07	0.341
MCV (fL)	0.014	1.01	0.96–1.06	0.580	0.020	1.02	0.98–1.07	0.356
MCH (pg)	0.023	1.02	0.89–1.16	0.718	0.015	1.02	0.92–1.13	0.770
PLT (*10^9^)	0.002	1.00	0.99–1.01	0.439	0.000	1.00	0.99–1.01	0.959
MPV (fL)	0.136	1.15	0.88–1.51	0.320	0.039	1.04	0.70–1.56	0.847
WBC (*10^9^)	−0.153	0.86	0.71–1.03	0.105	0.041	1.04	0.92–1.19	0.525
LYM% (%)	0.013	1.01	0.99–1.06	0.959	0.002	1.00	0.97–1.03	0.890
LYM# (*10^9^)	0.026	1.03	0.61–1.70	0.144	0.074	1.08	0.78–1.50	0.655
Biochemical parameters								
ALT (U/L)	0.027	1.03	0.99–1.07	0.119	0.014	1.01	0.97–1.07	0.561
AST (U/L)	0.030	1.03	0.99–1.08	0.180	0.005	1.00	0.98–1.03	0.670
BUN (mmol/L)	−0.252	0.78	0.61–0.99	0.044*	−0.330	0.72	0.55–0.93	0.012*
CREA (umol/L)	−0.021	0.98	0.95–1.00	0.096	0.003	1.00	0.98–1.03	0.823
TBIL (umol/L)	0.043	1.04	0.91–1.19	0.531	0.026	1.03	0.97–1.09	0.345
DBIL (umol/L)	0.161	1.17	0.79–1.75	0.427	0.137	1.15	0.97–1.37	0.117
IBIL (umol/L)	0.051	1.05	0.87–1.28	0.605	0.017	1.02	0.96–1.09	0.599

*SpO*_*2*_ blood oxygen saturation, *HR* heart rate at resting, *SBP* systolic blood pressure, *DBP* diastolic blood pressure, *RBC* red blood cell count, *Hgb* hemoglobin, *Hct* hematocrit, *MCV* mean corpuscular volume, *MCH* mean corpuscular hemoglobin, *PLT* platelets count, *MPV* mean platelet volume, *WBC* white blood cell, *LYM%* lymphocyte rate, *LYM#* lymphocyte count, *ALT* alanine aminotransferase, *AST* aspartate aminotransferase, *BUN* blood urea nitrogen, *CREA* creatinine, *TBIL* total serum bilirubin, *DBIL* direct serum bilirubin, *IBIL* indirect serum bilirubin

*p value indicates *p* < 0.05; **p value indicates *p* < 0.01

Multivariate logistic regression was performed for parameters which showed significant associations with HAH in univariate logistic regression, correlated with headache score, or showed significant difference between HAH+ and HAH- groups. For parameters at sea level, only BUN showed significant protective effect on HAH (OR:0.77, 95% CI:0.60–0.99, *p* = 0.040), but for parameters at 3700 m, multivariate logistic regression revealed that SpO_2_ (OR:0.84, 95% CI:0.76–0.93, *p* < 0.001), HR (OR:1.03, 95% CI:1.00–1.07, *p* = 0.042) and BUN (OR:0.64, 95% CI:0.46–0.88, *p* = 0.007) were all independent risk factors for HAH (Table 5).

**Table 5:** Forward stepwise multivariate logistic regression for HAH at sea level and 3700 m (*N* = 318)

	β-coefficient	OR	95% CI	*p*-value
Measurements at sea level (after variable selection)				
age	0.075	1.08	0.99–1.18	0.092
BUN	−0.259	0.77	0.60–0.99	0.040*
Measurements at 3700 m (after variable selection)				
age	0.090	1.09	0.98–1.23	0.110
SpO_2_	−0.191	0.84	0.76–0.93	0.001**
HR	0.029	1.03	1.00–1.07	0.042*
SBP	−0.019	0.98	0.95–1.01	0.091
BUN	−0.452	0.64	0.46–0.88	0.007**

*SpO*_*2*_ blood oxygen saturation, *HR* heart rate at resting, *SBP* systolic blood pressure, *BUN* blood urea nitrogen

*p value indicates *p* < 0.05; **p value indicates *p* < 0.01

## Discussion

### Alterations in physiological, hematological and biochemical parameters

Our study identified alterations in physiological, hematological and biochemical parameters from sea level to altitude of 3700 m. High altitude hypoxia lead a reduction of SpO_2_, which may result in a decrease in the delivery of oxygen and energy to organs and tissues. Dropped blood oxygen level may stimulate carotid chemoreceptors and activate the autonomic nervous system, which results in the cardiac output improvement, finally leading to increased HR [20]. The observation of our study is in consistency with previous studies [1, 7, 13].

The low humidity, hypoxic tachypnea and reduced fluid intake can lead to insensible fluid lose at high altitude [15, 16]. In addition, within hours of exposure to high altitude hypoxia, hypoxic tachypnea may lead to respiratory alkalosis, increased natriuresis and diuresis, promoting fluid shift away from intravascular space, result in blood concentration, even hypovolemia [21]. Our study observed most hematological parameters (RBC, Hgb, Hct, MCH, PLT, WBC and LYM#) were elevated from sea-level to altitude of 3700 m within a 72 h ascending processes, which also in consistency with previous studies [14].

**Fig. 2:**
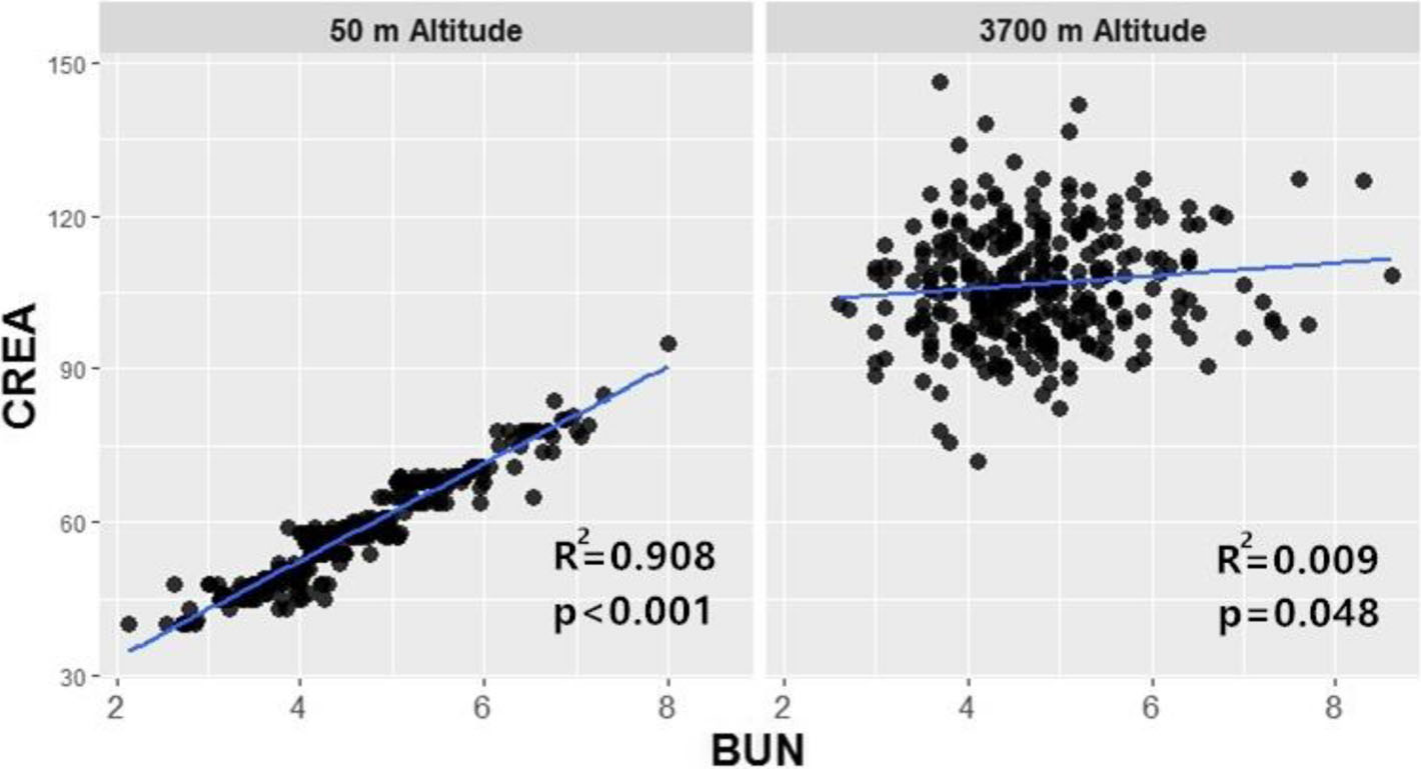
the correlation between BUN and creatinine at 50 m and 3700 m. BUN, Blood urea nitrogen (mmol/L); CREA, creatinine (umol/L)

Our study also observed that serum creatinine increased dramatically after ascent from sea level to 3700 m, indicating a significantly decreased estimated glomerular filtration rate (eGFR). Previous study has suggested that a linear decrease of eGFR with the increase of altitude, which may related to a reduction of renal plasma flow (secondary to the increased sympathetic activity) [18]. In addition, our study found that there was only little change of BUN from sea level to 3700 m, which is inconsistent with previous study [22]. Because both BUN and creatinine were commonly used as renal function markers, we draw a scatter plot to explore the change of the relationship between BUN and creatinine at 50 m and 3700 m (See fig. 2). This figure showed that there is a strong linear correlation between BUN and creatinine is at 50 m altitude, but no significant correlation after ascent to 3700 m. The BUN level represent the urea concentration of plasma, which can be reabsorbed in inner medullary collecting duct (IMCD), while creatinine can not be reabsorbed [23, 24]. Some studies indicated that urea generation in hepatocytes can be obstructed by insufficient adenosine triphosphate (ATP) supply and depressed levels of arginine and citrulline after exposure to hypoxia, but the genesis of creatinine is relatively constant [25]. This may be an explanation to the change of relationship between BUN and creatinine after rapid ascending to high altitude.

### The physiological risk factors for HAH at 3700 m

The elevation of altitude results in a lower partial pressure of oxygen in the inspired air, and SpO_2_ is a direct parameter that reflect the oxygen delivery. Insufficient oxygen consumption of cerebral tissue leads to function disorder and cytotoxic oedema, which is the main cause of HAH [26]. In addition, increased HR reflect the activity of sympathetic nervous system, which can lead to higher cardiac output and vasoconstriction of viscera, promote the redistribution of blood (mostly into vital organs such as brain). The accumulation of fluid in the brain result in increased intracranial pressure (ICP), which is another crucial cause of HAH [6]. Multiple lines of studies have reported that reduced SpO_2_ and increased HR are independent risk factors for HAH, which is supported by our findings [1, 7, 11].

### Blood urea nitrogen is an independent risk factor for HAH at both sea level and 3700 m

Our results first found that BUN is an independent risk factor of HAH at both sea level and 3700 m, and the values of BUN at 3700 m positively associated with HAH severity. Although more evidence of this finding is lacking, there still can be some potential explanations.

The first possible explanation is that the urea concentration may reflect the oxygen supply and utilization in cells. As discussed above, the correlation between BUN and creatinine showed a good linearity at sea level but no correlation at 3700 m, suggest that the production of BUN may be affected by high altitude environment, and the concentration of BUN may partially reflect metabolism status and the oxygen utilization status of hepatocyte. Relative to BUN, other liver function parameters such as ALT, AST and bilirubin were aimed at substantial damage of liver and biliary tract. Some vitro experiments demonstrated that under hypobaric hypoxia, ATP was decreased in multiple cell lines, and as the oxygen concentration was decreased, production of both urea in isolated rat hepatocytes declined [25, 27, 28]. Study on the effect of acute hypoxic hypoxia on the profile of plasma amino acids in rats showed that after exposure to hypoxia for 5 h, the concentrations of arginine and citrulline (which are related to the urea cycle) were depressed [25]. These findings implicate that the BUN concentration in plasma may reflect the oxygen-driven catabolism, and higher BUN may indicate better oxygen supply and utilization of hepatocytes, even brain cells, which is the main cause of HAH.

The second potential explanation is that the hyperosmolar properties of urea may help with reducing intracranial pressure and brain volume [29]. Through brain imaging of patients with acute mountain sickness, some studies showed that intracellular and extracellular water accumulation influenced by increased permeability of the blood–brain barrier, resulted in cerebral swelling, sulci disappearing and changing of grey matter [6]. These verified that the inflation of brain volume and elevation of ICP is a vital sign of HAH. In 1960s, urea became the first widely used hyperosmolar compound in clinic for reducing ICP and alleviating cerebral swelling [30, 31]. The penetrability of urea from extracellular into brain tissue is 1/10 compared with the penetration into muscle, and its blood to brain transfer coefficient (a measurement of clearance) is 5 × 10^− 3^ ml/g/min, a value that is 3 orders of magnitude lower than water [32]. Therefore, exogenous urea can maintain certain osmotic pressure inside and outside the brain cell and prevent excessive accumulation of liquids and cerebral swelling. Because urea undergoes renal excretion, the dehydrating effect of exogenous urea on parenchyma is short lived, and the effect of endogenous urea during brain oedema have not been studied.

Another possible explanation is the products from urea cycle, nitric oxide (NO). Arginine generated as intermediate products by argininosuccinate lyase during urea cycle, and is the basic substrate of nitric oxide synthase (NOS) for generating nitric oxide. Nitric oxide has a short half-life and rapidly diffuses into the vascular smooth muscle where it affects modulation of calcium ions, mediated by cyclic guanosine monophosphate (cGMP), leading to vasodilatation [33]. They have significant effects in relieving pulmonary hypertension, improve cardiac output and blood gas exchange [34]. However, relevant studies about the correlation between ICP, pulmonary hypertension and endogenic urea are still lacking.

### Limitations

Limited by field study, the time of onset of headache of participants were recorded by memories, not precisely, which should be improved in future. After the onset of headache, participants have not descent to low altitude immediately, which not strictly satisfied the criteria of the International Classification of Headache Disorders. The sample size was small, and there are many other potential risk factors that can be considered in the study, such as nitric oxide and PaCO_2_. The participants in our study were all young male individuals, which may limit extrapolation of our results.

## Conclusions

Our study found the frequency of HAH was high (74.84%) after acute exposure to 3700 m. We confirmed that SpO_2_ and HR at 3700 m are independent risk factors for HAH, and firstly identified the independent association between BUN and HAH at both sea level and 3700 m, which suggested that lower BUN may be a new independent risk factor for HAH.

## Additional files

Additional file 1: The distribution and QQ-norm plot of SpO2 at 50 m and 3700 m. (DOCX 168 kb)

Additional file 2: The incidence of mild, moderate and severe headaches after ascent to 3700 m altitude. (DOCX 16 kb)

Additional file 3: The Shapiro-Wilk normality test of parameters at 50 m and 3700 m. (DOCX 12 kb)

## Abbreviations

ALT: alanine aminotransferase
AMS: acute mountain sickness
AST: aspartate aminotransferase
ATP: adenosine triphosphate
BMI: body mass index
BUN: blood urea nitrogen
cGMP: cyclic guanosine monophosphate
CREA: creatinine
CRFs: Structured case report forms
DBIL: direct serum bilirubin
DBP: diastolic blood pressure
eGFR: estimated glomerular filtration rate
HAH: high-altitude headache
Hct: hematocrit
Hgb: hemoglobin
HR: heart rate
IBIL: indirect serum bilirubin
ICP: intracranial pressure
IMCD: inner medullary collecting duct
IQR: interquartile range
LYM: absolute lymphocyte count
LYM%: lymphocyte percentage
MCH: mean corpuscular hemoglobin
MCV: mean corpuscular volume
MPV: mean platelet volume
NO: nitric oxide
NOS: nitric oxide synthase
OR: Odds ratio
PLT: platelet count
RBC: red blood cell count
SBP: systolic blood pressure
SD: standard deviations
SpO2: blood oxygen saturation
TBIL: total serum bilirubin
WBC: white blood cell count
