# Supplementary material for: Physiological, hematological and biochemical factors associated with high-altitude headache in young Chinese males following acute exposure at 3700 m
Source: J Headache Pain. 2018 Jul 25;19(1):59. doi: 10.1186/s10194-018-0878-7 (PMC6060196; doi:10.1186/s10194-018-0878-7)
Supplement: Supplementary file 1 — The distribution and QQ-norm plot of SpO2 at 50 m and 3700 m. (DOCX 168 kb) [file 10194_2018_878_MOESM1_ESM.docx]

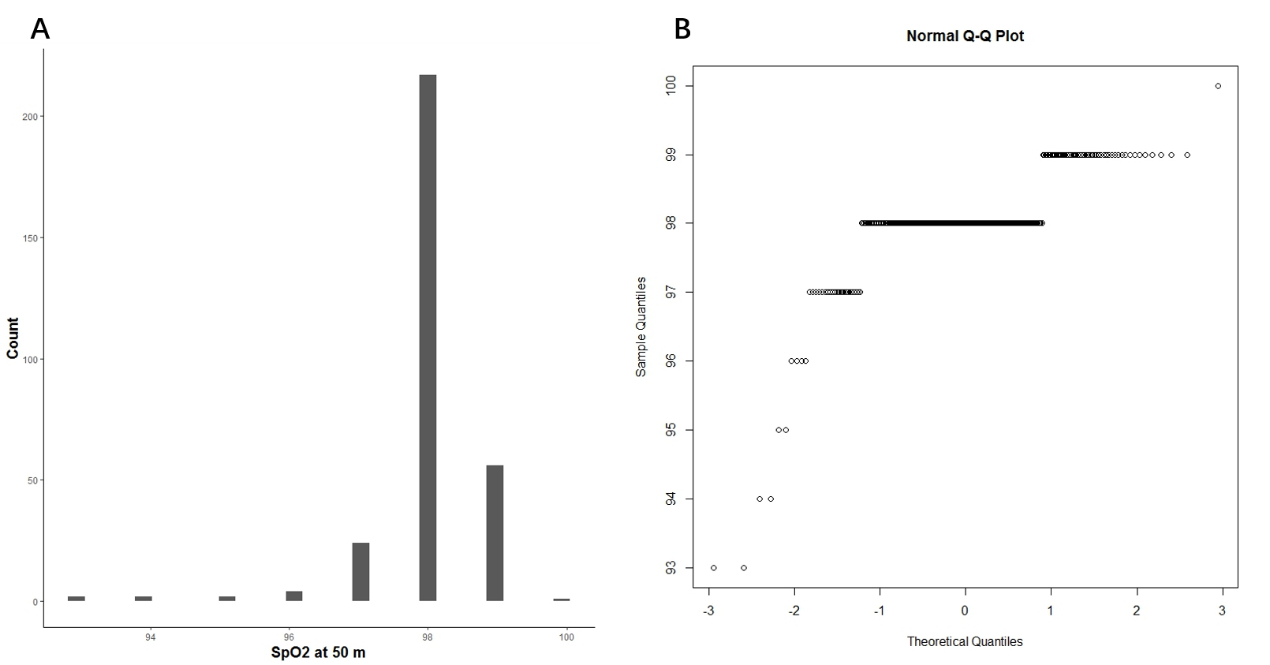


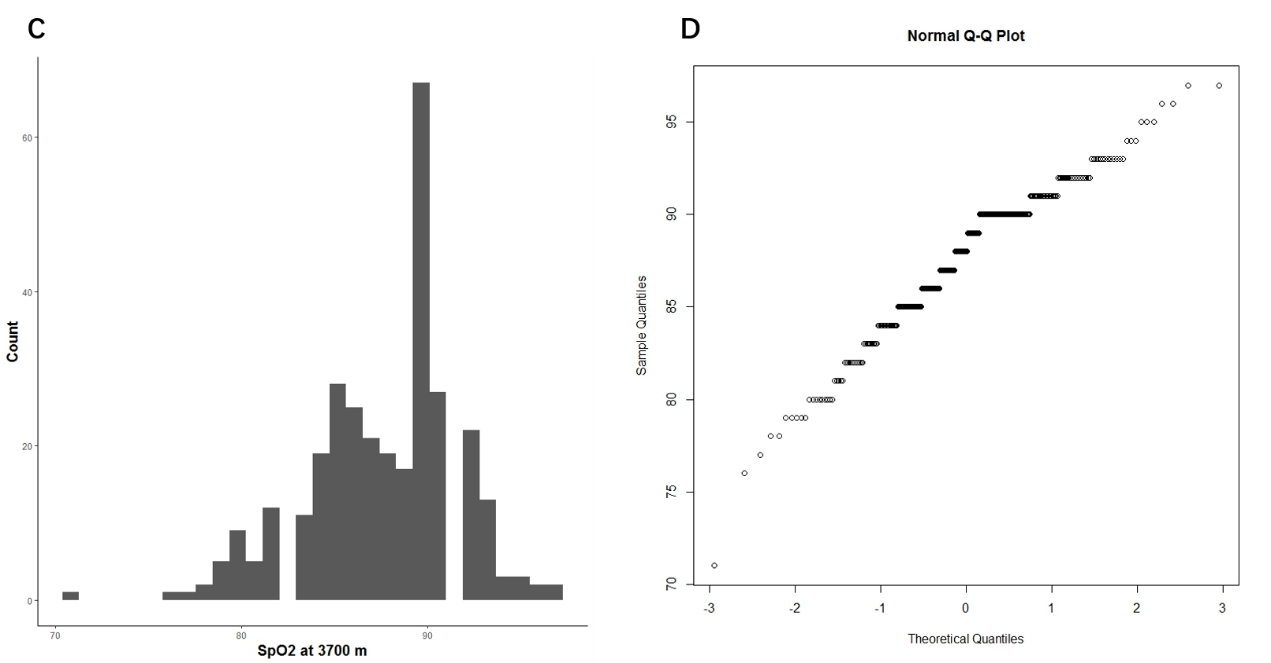


**Supplementary Figure 1**. The distribution and QQ-norm plot of SpO2 at 50 m and 3,700 m.

1. The distribution of SpO2 at 50 m altitude
2. The QQ-norm plot of SpO2 at 50 m altitude
3. The distribution of SpO2 at 3,700 m altitude
4. The QQ-norm plot of SpO2 at 3,700 m altitude
