# Supplementary material for: Physiological, hematological and biochemical factors associated with high-altitude headache in young Chinese males following acute exposure at 3700 m
Source: J Headache Pain. 2018 Jul 25;19(1):59. doi: 10.1186/s10194-018-0878-7 (PMC6060196; doi:10.1186/s10194-018-0878-7)
Supplement: Supplementary file 2 — The incidence of mild, moderate and severe headaches after ascent to 3700 m altitude. (DOCX 16 kb) [file 10194_2018_878_MOESM2_ESM.docx]

Supplementary table 2. The Shapiro-Wilk normality test of parameters at 50 m and 3,700 m

|  |  | **Measurements at sea level** | |  | **Measurements at 3,700 m** | |
| --- | --- | --- | --- | --- | --- | --- |
|  |  | W | p |  | W | p |
| **Demographic data** |  |  |  |  |  |  |
| Age (year) |  | 0.852 | <0.001 |  |  |  |
| BMI (kg/m**^2^**) |  | 0.977 | <0.001 |  |  |  |
| Chest Circumstance (cm) |  | 0.983 | <0.001 |  |  |  |
| **Physiological parameters** |  |  |  |  |  |  |
| SpO_2_ (%) |  | 0.455 | <0.001 |  | 0.962 | <0.001 |
| HR (beats/min) |  | 0.991 | 0.061 |  | 0.99 | 0.03 |
| SBP (mmHg) |  | 0.993 | 0.128 |  | 0.991 | 0.131 |
| DBP (mmHg) |  | 0.993 | 0.187 |  | 0.985 | 0.014 |
| **Hematological parameters** |  |  |  |  |  |  |
| RBC (*10^12^) |  | 0.952 | <0.001 |  | 0.949 | <0.001 |
| Hgb (g/L) |  | 0.987 | 0.007 |  | 0.904 | <0.001 |
| Hct (%) |  | 0.984 | 0.002 |  | 0.921 | <0.001 |
| MCV (fL) |  | 0.829 | <0.001 |  | 0.779 | <0.001 |
| MCH (pg) |  | 0.754 | <0.001 |  | 0.807 | <0.001 |
| PLT (*10^9^) |  | 0.973 | 0.231 |  | 0.99 | 0.058 |
| MPV (fL) |  | 0.985 | 0.017 |  | 0.977 | <0.001 |
| WBC (*10^9^) |  | 0.912 | <0.001 |  | 0.941 | <0.001 |
| LYM rate (%) |  | 0.907 | 0.648 |  | 0.991 | 0.074 |
| LYM count (*10^9^) |  | 0.958 | <0.001 |  | 0.962 | <0.001 |
| **Metabolic parameters** |  |  |  |  |  |  |
| ALT (U/L) |  | 0.830 | <0.001 |  | 0.886 | <0.001 |
| AST (U/L) |  | 0.901 | <0.001 |  | 0.683 | <0.001 |
| BUN (mmol/L) |  | 0.979 | <0.001 |  | 0.831 | <0.001 |
| Creatinine (umol/L) |  | 0.964 | <0.001 |  | 0.634 | <0.001 |
| TBIL (umol/L) |  | 0.955 | <0.001 |  | 0.451 | <0.001 |
| DBIL (umol/L) |  | 0.912 | <0.001 |  | 0.579 | <0.001 |
| IBIL (umol/L) |  | 0.957 | <0.001 |  | 0.633 | <0.001 |
