# Supplementary material for: Physiological, hematological and biochemical factors associated with high-altitude headache in young Chinese males following acute exposure at 3700 m
Source: J Headache Pain. 2018 Jul 25;19(1):59. doi: 10.1186/s10194-018-0878-7 (PMC6060196; doi:10.1186/s10194-018-0878-7)
Supplement: Supplementary file 3 — The Shapiro-Wilk normality test of parameters at 50 m and 3700 m. (DOCX 12 kb) [file 10194_2018_878_MOESM3_ESM.docx]

Supplementary table 1.The incidence of mild, moderate and severe headaches after ascent to 3,700 m altitude

|  | **Headache Severity** | | | | | | |
| --- | --- | --- | --- | --- | --- | --- | --- |
|  | **Non Headache** |  | **Mild Headache** |  | **Moderate Headache** |  | **Severe Headache** |
| **Number** | 80 |  | 193 |  | 42 |  | 3 |
| **Percent** | 25.16% |  | 60.69% |  | 13.21% |  | 0.94% |
